# Supplementary material for: Out of control mortality matters: the effect of perceived uncontrollable mortality risk on a health-related decision
Source: PeerJ. 2014 Jun 26;2:e459. doi: 10.7717/peerj.459 (PMC4081279; doi:10.7717/peerj.459)
Supplement: Supplemental Information 1 — This supplement contains additional detail on the methods used in the experiments reported in the Pepper & Nettle paper, “Out of control mortality matters: the effect of perceived uncontrollable mortality risk on a health-related decision.” The supplementary information includes: (1) Details on the methods used to ensure the data used were from participants who had been honest about their location. (2) The participant information, consent, questionnaire and debrief used in experiments 1 and 2, and (3) the prize draw cards used in experiment 3. [file peerj-02-459-s001.pdf]

Supplement: Supporting information on methods for Pepper & Nettle, "Out of control mortality matters: the effect of perceived uncontrollable mortality risk on a health-related decision"

This supplement contains additional detail on the methods used in the experiments reported in the Pepper & Nettle paper, "Out of control mortality matters: the effect of perceived uncontrollable mortality risk on a health-related decision." The following supplementary information is included:

- Details on the methods used to ensure the data used were from participants who had been honest about their location (p1-2).
- The participant information, consent, questionnaire and debrief used in experiments 1 and 2 (p3-6).
- The prize draw cards used in experiment 3 (p7-9).

Participant location checks for experiments 1 and 2

We needed to ensure that our participants were from the UK, because the primes were based on UK postcode statistics. Thus, when we requested participants through Crowdflower, we made UK residency a criterion. However, the Crowdflower system relied on participant honesty to ensure that they fulfilled this condition. Therefore, we took additional measures to ensure that the data that we used came from participants who had been honest about their location. After completing the consent form, participants were moved onto a location-check screen, which read "Your IP address indicates that you are currently in [LOCATION]." Qualtrics was programmed to present the location from which the participant was accessing the web page, based on their Internet Protocol address (IP address). The rest of the screen read, "We only need people who live in the UK to complete our survey. However, we understand that you may be taking the survey whilst away from home. If you are not from the UK we can still generate a code so you get credit for trying to take part, but we will not be able to use your answers as part of our study. Do you currently live in the UK?" Participants then had the option to click "Yes" or "No." Participants who clicked "no" were filtered straight to the debriefing screen and were given Crowdflower credit for their time. Participants who declared themselves to be from the UK were moved on to the rest of the experiment.

Experiment 1 - exclusions based on location

We requested 100 responses through Crowdflower. However, we were compelled to exclude some of the data. A key element of the prime was that the mortality risk information was tailored to participants' postcodes. Therefore, we only used data for which we could be reasonably certain that the participants had entered their real postcode. We excluded data from our analysis if: 1) The participant's postcode was missing or was not a valid UK postcode, 2) the participant's IP address was not UK based, or 3) the participant IP address recorded by Crowdflower did not match that recorded by Qualtrics (indicating possible use of a proxy server or an attempt to take the same survey

multiple times from different machines in order to get extra Crowdflower credit.) After these exclusions we were left with 72 responses.

Experiment 2 - exclusions based on location

The exclusion criteria were the same as those used in experiment 1 (above). Since both experiments 1 and 2 used Crowdflower as a recruitment platform, we used both IP addresses and postcode, age and gender combinations to check that participants in experiment 2 had not previously taken part in experiment 1. No repeat participants were identified. After exclusions, we were left with a sample of 195 participants.

Experiments 1 & 2 – participant information, consent, questionnaire and debrief

Participant Information

The UK health behaviours study

Welcome to the UK health behaviours study. We want to understand why people in some parts of the UK live longer than others do. (For recent information about differences in life expectancies across the UK, please see this BBC news article.)

What is involved?

We will ask you for your age, gender and location (postcode). Then we will ask you some questions about your health habits. You must be over 18 and living in the UK to take part. The whole survey should take no more than 5 minutes. Participation is voluntary and you are free to withdraw from the study at any time without giving a reason. The study has ethical approval from the Newcastle University Faculty of Medical Sciences (ref: 00554). If you have any concerns about the way the study was conducted please contact either Prof Daniel Nettle (daniel.nettle@ncl.ac.uk) or the ethics committee (fmsethics@newcastle.ac.uk).

What happens to the information gathered?

The information recorded for the study is anonymous and will be kept on password-protected computers, or in a locked filing cabinet at the University. Participants will not be personally identifiable in any research papers arising from this study. We will not share your information with any third parties.

Questions?

If you have any questions about the study please contact the lead researcher (below) by email or post.

Gillian Pepper, Henry Wellcome Building, Newcastle University, NE2 4HH

Email: g.pepper@ncl.ac.uk

Thank you!

Timing recorded

First Click (1)

Last Click (2)

Page Submit (3)

Click Count (4)

Consent

Please click next to each of the following statements to indicate that you understand them and wish to take part in the study. Then click ">>" to continue.

☐ I have read and understood the information provided for the study and have seen the email address I can contact to ask questions about it. (1)

☐ I understand that participation in this study is entirely voluntary and I can withdraw at any time without giving any reason and without any of my rights being affected. (2)

☐ I understand that all electronic data created during the study will be stored in anonymised form on password-protected computers. (3)

☐ I understand that all the information will be treated as confidential, and that I will not be personally identified in any way. (4)

☐ I confirm that I wish to take part in the study. (5)

Location information

Your IP address indicates that you are currently in Newcastle Upon Tyne. We only need people who live in the UK to complete our survey. However, we understand that you may be taking the survey whilst away from home. If you are not from the UK we can still generate a code so you get credit for trying to take part, but we will not be able to use your answers as part of our study. Do you currently live in the UK? Select your answer then click ">>" to continue.

☐ Yes (1)

☐ No (2)

[If No Is Selected, Then Skip To End of Survey]

Demographic questions

How old are you?

[Drop-down menu, age options 18-100]

Are you male or female?

☐ male (1)

☐ female (2)

What is your current postcode?

[Text box]

Processing screen

Thanks for submitting your information. It may take a while to match it to health data for people of your age and gender in your postcode area. Please wait a few moments. Thank you.

Timing recorded

First Click (1)

Last Click (2)

Page Submit (3)

Click Count (4)

[Random allocation to primes – see manuscript for prime text]

Timing recorded

First Click (1)

Last Click (2)

Page Submit (3)

Click Count (4)

Health intention questions

How much effort will you put into looking after your health and safety over the coming week? 0 means no effort at all, 100 means the maximum effort you could make.

[Sliding scale 0-100]

How likely is it that you will eat 5 portions of fruit or vegetables per day over the coming week? 0 means you definitely won't eat 5 portions of fruit and veg per day, 100 means you definitely will eat 5 portions of fruits or veg per day.

[Sliding scale 0-100]

How likely is it that you will do 30 minutes or more of physical exercise three times over the coming week? 0 means you definitely won't do three 30-minute blocks of exercise, 100 means you definitely will.

[Sliding scale 0-100]

How many units of alcohol are you likely to drink over the coming week. (One standard glass of wine or pint of lager is about 2.3 units.) If you are unsure about how many units of alcohol are in different drinks, you can calculate them here: NHS Units Calculator

[Text box]

Prize draw question

Thanks for taking part in our study. To say thank you, we would like to enter you into a prize draw. There are two choices of prize available. Please select the prize you would prefer to win, then enter your email address or telephone number in the box below, so that we can contact you to organize delivery if you win.

Privacy: We will not use your contact details for any other purpose, or pass them to any third parties. Your contact details will be deleted from our records once the prize draw has taken place.

What would you prefer to win?

☐ A Riverford Farm Organic Fruit box worth £11. (1)

☐ A Thorntons Summer Chocolate Collection box worth £11. (2)

Contact If you win the prize draw, what is your preferred contact email address or phone number?

[Text box]

End of survey debrief message

The health behaviours study

Thanks for taking part in this study. If you are not living in the UK we could not include you in the study, but thanks for trying to take part. You will still be awarded credit.

Your validation code for Crowdflower is [insert code]

The statistics we presented to you about people in your area were false. We did not tell you the true purpose of the study because we wanted you to answer our questions as honestly as possible.

The experiment was actually about whether changing people's ideas about how long they might live alters their motivation to look after their health. You will have been told that people of your age, living in your area, are living longer than others in the UK, or that they are dying younger than others in the UK are. This was not real information.

We expect the experiment to have had a short-term influence on your motivation to look after your own health. However, this effect should not be long lasting. If you feel that you have been unduly affected by your experience of this study, please contact Prof Daniel Nettle on +44 (0)191 222 8993 or daniel.nettle@ncl.ac.uk

If you want to know more about our reasons for running study you might wish to read the following paper:

Nettle, D. (2010). Why are there social gradients in preventative health behavior? A perspective from behavioral ecology PLoS ONE 5(10): e13371: Link to paper PDF

If you have any questions about the study please email g.pepper@ncl.ac.uk

The prize draw cards used in experiment 3

Personal details box – used in both treatments

PRIZE DRAW!

3 x £100 gift cards to be won

HOW TO ENTER:

1. Circle your answer to the question overleaf.
2. Enter your name, address and postcode in the box provided opposite.
3. Put your card into the correct box for your preferred bonus prize (details opposite).

Please do not enter more than once. Repeat entries will be discarded. **You must be over 18 to enter.**

Newcastle University

ESSENTIALS:

Name:

Address:

Postcode:

Date of birth:

OPTIONAL: Want us to send you more information about the study? Write your email address here:

10 x BONUS PRIZES! CHOOSE BETWEEN FRUIT AND CHOCOLATE

Would you prefer to win a Riverford Farm Organic Fruit box worth £11, or a Thorntons Chocolate Collection box worth £11? Tell us by posting this card into the corresponding box.

BONUS PRIZE:

CHOICE NUMBER 1

Riverford Farm Organic Fruit Box

BONUS PRIZE:

CHOICE NUMBER 2

Thorntons Chocolate Collection Box

Controllable long life priming card

PRIZE DRAW!

3 x £100 gift cards to be won

HOW TO ENTER:

1. Circle your answer to the question opposite.
2. Enter your name, address and postcode in the box provided overleaf.
3. Put your card into the correct box for your preferred bonus prize (details overleaf).

Please do not enter more than once. Repeat entries will be discarded. **You must be over 18 to enter.**

10 x BONUS PRIZES!

Choose fruit or chocolate boxes

Newcastle University

Our question: Recent statistics show that people in Tyne and Wear are living longer now than they were in the year 2000. Why do you think this is?

A) Because people have more control over the kind of healthcare they receive.

B) Because people are looking after themselves better.

C) Both: people have more control over their care **and** are looking after themselves better.

This study has received ethical approval from the Newcastle University Faculty of Medical Sciences ethics committee (reference: 00653/2013). By completing this card you are giving your consent for your answers to be used in our study. **We will not share your contact details with anyone else. We will only use them to deliver your prize to you if you win.**

Uncontrollable long life priming card

PRIZE DRAW!

3 x £100 gift cards to be won

HOW TO ENTER:

1. Circle your answer to the question opposite.
2. Enter your name, address and postcode in the box provided overleaf.
3. Put your card into the correct box for your preferred bonus prize (details overleaf).

Please do not enter more than once. Repeat entries will be discarded. **You must be over 18 to enter.**

10 x BONUS PRIZES!

Choose fruit or chocolate boxes

Newcastle University

Our question: Recent statistics show that people in Tyne and Wear are living longer now than they were in the year 2000. Why do you think this is?

A) Because there are fewer traffic accidents.

B) Because there is less violent crime.

C) Both: there are fewer traffic accidents **and** less violent crime.

This study has received ethical approval from the Newcastle University Faculty of Medical Sciences ethics committee (reference: 00653/2013). By completing this card you are giving your consent for your answers to be used in our study. **We will not share your contact details with anyone else. We will only use them to deliver your prize to you if you win.**
